# Laboratory study on the deformation resistance indicators of asphalt mixture based on the rutting deformation growth model

**DOI:** 10.1371/journal.pone.0340720

**Published:** 2026-02-12

**Authors:** Lihua Liu, Zhaoyu Sun

**Affiliations:** School of Civil and Transportation Engineering, Henan University of Urban Construction, Pingdingshan City, China; China Construction Fourth Engineering Division Corp. Ltd, CHINA

## Abstract

Rutting tests were conducted to investigate the deformation resistance of asphalt mixtures under varying temperature and wheel load conditions. The significant effect of temperature and load on the deformation resistance of the asphalt mixture was investigated using ANOVA. Multiple deformation resistance indicators, including complex stability index (*CSI*), densification coefficient (D), shear index(1/*E*), and characteristic parameters (*A*, *C*, and *A/C*), were proposed based on the rutting deformation growth equation, and the correlation model between rutting deformation and each indicator was established. The validity and reliability of the deformation resistance indicators were verified and evaluated. The results demonstrated that both the dynamic stability and rutting deformation of the asphalt mixture are more sensitive to temperature than load at the 0.05 significance level. The correlation between the proposed indicators and rutting deformation were significantly stronger than that between dynamic stability and rutting deformation. Additionally, indicators *A* and *A*/*C* exhibit high level of correlation when predicting permanent rutting deformation. The proposed deformation resistance indicators can accurately predict the permanent rutting deformation in laboratory rutting tests, which can provide reference for studying long-time rutting deformation in laboratory rutting tests.

## 1. Introduction

With the rapid development of economy and society over the past three decades, the road traffic volume has increased dramatically, leading to higher demands for pavement performance, particularly in terms of capacity and durability. Asphalt pavements, owing to its advantages such as good crack resistance, convenient construction, and cost-effectiveness, has become the predominant choice for expressways worldwide. In China, more than 90% of pavement used asphalt layers as the surface course. The typical forms of damage for asphalt pavements are rutting and cracking. Rutting refers to the longitudinal surface depression that occurs on the wheel paths of road due to shear and compressive stresses under repetitive traffic loading [1]. Rutting deformation is one of the most critical early diseases of asphalt pavement, which not only reduces the smoothness of the pavement and increases driving risks, but also affects the road performance and service life seriously [2]. However, as rutting is still a major disease for asphalt pavement, it is necessary to evaluate the rutting resistance of the materials used in each structural layer of asphalt pavement, to improve the safety and comfortability of the pavement.

Extensive research has been conducted to evaluate the rutting resistance of asphalt mixtures by different test methods were utilized to estimate the rutting performance of asphalt pavement [3–9]. And most of the previous research focused on the evaluation and improvement of the rutting resistance [10–13]. Some researchers developed rutting prediction models by employing several machine learning techniques such as Artificial Neural Network, regression tree, support vector machine, ensembles, Gaussian process regression, and Deep learning techniques [14–18]. However, these studies did not address the deformation resistance indicator. The Chinese specification of JTG F40-2004 [19] recommends dynamic stability (DS), which is determined by wheel tracking tests, as the indicator for evaluating the rutting deformation of asphalt mixture. Du and Dai examined the effects of both the DS index and the complex stability index (*CSI*) on the stiffness modulus of asphalt mixture, which demonstrating that the *CSI* serves as a more effective indicator of rutting deformation [20].

These indicators that characterize rutting performance can be broadly divided into two categories namely, direct rutting indicators and indirect rutting indicators. The direct rutting indicators can be obtained through laboratory tests, such as the rutting test, while the indirect rutting indicators require either repeated loading tests or further analysis of test data. Despite these efforts, most existing indicators fall short in predicting long-term rutting performance, especially under varying environmental and loading conditions.

Variations of material properties are the main factors that affect the rutting resistance of asphalt mixtures [21,22], while environmental factors, mainly temperature, and vehicle load have a significant effect on material properties [23]. Therefore, temperature and vehicle load are the main factors affecting rutting resistance. The accumulation of permanent deformation under repeated load is a function of temperature, vehicle load and velocity [24–27]. Higher temperature, heavy load and lower velocity result in greater permanent deformation. The laboratory rutting test is loaded at a uniform speed (42 times/min), so the effect of loading speed on deformation is not considered in laboratory test.

As one of the most important factors, temperature has greatly influenced on the performance of asphalt pavements [28]. This is because that the viscosity of asphalt decreases with the temperature increases, especially when the temperature exceeds the softening point of asphalt, which resulting in a reduction in the deformation resistance of the mixture [29]. In addition, the degree of aging and fatigue properties of pavement materials are also affected by temperature, which affects the deterioration of rutting. Previously study shows that temperature of asphalt pavement can exceed 60 °C when the air temperature reaches more than 40 °C [30]. When there is continuous high temperature weather, rutting damage will occur on asphalt pavement, and the rutting deformation develops rapidly. Pouranian et al investigated the influence of temperature and stress level on the rutting performance of modified stone matrix asphalt (SMA), and revealed that the stress sensitivity of rutting performance decreases with temperature increase [31]. Souza, F. V. et al used numerical simulations to study the combined effect of temperature and loading on the mechanical response of asphalt pavements, and the results showed that temperature and thermo-viscoelasticity must be considered in asphalt pavements design [32]. Wasage, T. L. J. et al investigated the rutting behaviors of different paving mixtures used the rutting test, and the results showed that the rutting deformation deepens with the increase of test temperature [33].

Vehicle load is another important factor that causes rutting deformation. Along with the increase of traffic volume, the proportion of overloaded vehicles and heavy-duty vehicles has increased, and the tire load of these vehicles is higher than the standard axle load (0.7MPa), or even more than 1.0 MPa, resulting in rapid deformation of the road surface [25,34,35]. Furthermore, the stress distribution and deformation pattern of pavement materials are influenced by vehicle load, which affects the deterioration degree of rutting.

Although researchers have put forward several indicators of rutting deformation resistance for asphalt mixture especially at the mixture proportion design. These indicators can reflect the performance of asphalt mixtures with conditions of different temperatures and loads, which providing a foundation for engineering design and material selection. However, most of these indicators reflect short-term rutting deformation, and current research lacks indicators for long-term rutting deformation. This paper aims to establish indicators for long-term rutting deformation through laboratory testing.

The surface structure of asphalt pavements is typically divided into the upper layer, middle layer, and lower layer. Different asphalt mixtures are used for each layer based on their functional requirements, and AC-13, AC-20, and ATB-30 are commonly employed for the surface layers of pavements. Therefore, in this paper, the AC-13, AC-20 and ATB-30 were taken as research objects to investigate deformation resistance indicators. Firstly, the relationship between deformation resistance of asphalt mixture and temperature and load were studied. Secondly, deformation resistance indicators are proposed based on rutting tests conducted under different temperatures and loads. Thirdly, the applicability of deformation resistance indicators was investigated and their effectiveness and reliability were compared. These indicators can be used to predict long-term rutting deformation. Investigating the objective law between rutting deformation and temperature and load can better understand the mechanical characteristics and deformation law of pavement structure, and provide a basis for pavement structure optimization design and construction. It can also provide reference for road maintenance and management, which is conducive to improving the performance of the road and extending the service life.

## 2. Materials and test methods

### 2.1. Materials

#### 2.1.1. Asphalt.

In this study, the 70# heavy traffic road petroleum asphalt was used, which is manufactured in Shangqiu, Henan province, China. Table 1 presents the general specifications of asphalt. The test methods can refer to Chinese specification of JTG 3410−2025 [36]. All the indexes comply with Chinese specification of JTG F40-2004 [19].

**Table 1:** General specifications of asphalt.

Material properties		Testing value	Code values	Test method
Penetration (25^o^C, 100 g, 5 s) (0.1 mm)		67	60-80	T 0604
Ductility (5 cm/min,15^o^C) (cm)		>100	≥100	T 0605
Ductility (5 cm/min,10^o^C) (cm)		39	≥25	T 0605
Softening point (^o^C)		47.5	≥46	T 0606
Density of bitumen(g/cm^3^,15^o^C)		1.036	Measured value	T 0603
Penetration index (PI)		−0.312	−1.5~+1.0	T 0604
Flash point (^o^C)		290	≥260	T 0611
Solubility in trichloroethylene (%)		99.6	≥99.5	T 0607
Paraffin content (%)		1.6	≤2.2	T 0615
Dynamic viscosity (Pa·s, 60^o^C)		213	≥180	T 0620
Thin-film heating test (163^o^C,5h)	Mass loss (%)	−0.3	−0.8~+0.8	T 0609
	Ductility (10^o^C) (cm)	7	≥6	T 0604
	Residual penetration Ratio(25^o^C) (%)	64	≥61	T 0605

#### 2.1.2. Aggregate.

The coarse and fine aggregate used in this study was crushed limestone gravel sourced from Shangqiu, Henan province, China. The main technical indicators of aggregate are tabulated in Table 2. All the test method can refer to Chinese specification of JTG E42-2005 [37].

**Table 2:** Technical indicators of aggregate.

Material properties	Aggregate size (mm)							Code values	Test method
	26.5-31.5	19-26.5	9.5-19	9.5-16	4.75-9.5	2.36-4.75	0-2.36		
Apparent density (g/cm^3^)	2.776	2.764	2.787	2.796	2.838	2.875	2.729	≥2.5	T 0304 T 0328
Ruggedness (%)	5.2	5.0	6.5	6.3	5.1	/	8.6	≤12	T 0314
Flakiness content (%)	6.7	7.3	6.5	6.3	5.1	/	/	≤20	T 0312
Moisture Absorption (%)	0.31	0.25	0.35	0.38	0.47	0.56	/	≤3.0	T 0304
Crushed stone value (%)	15.3	14.7	16.5	16.5	16.5	16.5	/	≤26	T 0317
Weared stone value (%)	14.9	15.2	16.1	18.1	18.1	18.1	/	≤28	T 0316
Aggregate angularity (s)	/					36.7		≥30	T0349
Methylene blue value (g/kg)	/					2.1		≤2.5	T 0349

#### 2.1.3. Mineral filler.

In the tests of this paper, the mineral filler was crushed limestone which sourced from Shangqiu, Henan province, China. The main technical indicators of mineral filler are shown in Table 3.

**Table 3:** Technical indicators of mineral filler.

Material properties	Testing value	Code values	Test method
Apparent density (g/cm^3^)	2.773	≥2.5	T 0352
Moisture content (%)	0.6	≤1.0	T 0359
Appearance	No agglomeration	No agglomeration	/
Hydrophilic coefficient (%)	0.6	≤1.0	T 0353
Plasticity index (%)	3.6	≤4.0	T0354

### 2.2. Aggregate gradation

**Fig 1:**
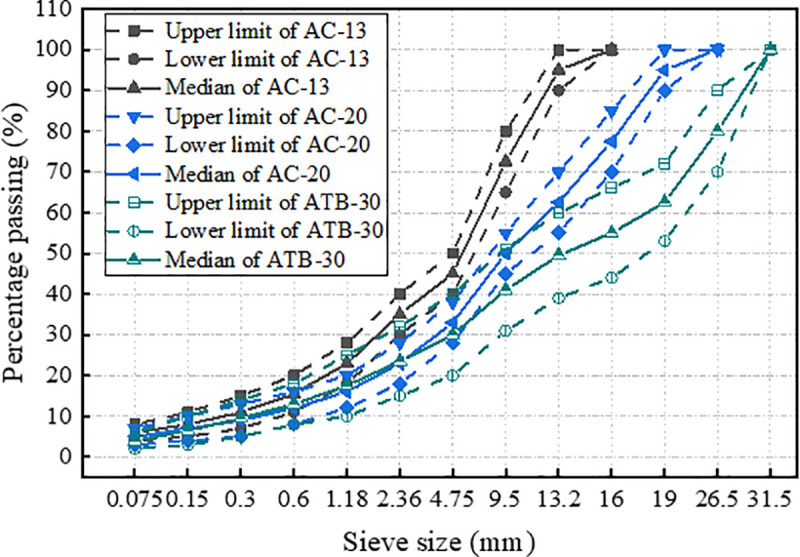
Mineral aggregate gradations of asphalt mixture.

Three aggregate gradations were used in this study, which are the asphalt mixture with a nominal maximum particle size of 13.2 mm (AC-13), 19 mm (AC-20), and 31.5 mm (ATB-30). The aggregate gradations are present in Table 4 and Fig 1, which simultaneously provides the upper limit and lower limit for all the asphalt mixtures.

**Table 4:** Aggregate gradations for asphalt mixture.

Mixture	Gradation	Passage rate (%) through the following sieves (mm)												
31.5	26.5	19	16	13.2	9.5	4.75	2.36	1.18	0.6	0.3	0.15	0.075
AC-13	Upper	–	–	–	100	100	80	50	40	28	20	15	11	8
	Lower	–	–	–	100	90	65	40	30	18	11	7	5	4
	Median	–	–	–	100	95	72.5	45	35	23	15.5	11	8	6
AC-20	Upper	–	100	100	85	70	55	38	28	20	16	13	10	7
	Lower	–	100	90	70	55	45	28	18	12	8	5	4	3
	Median	–	100	95	77.5	62.5	50	33	23	16	12	9	7	5
ATB-30	Upper	100	90	72	66	60	51	40	32	25	18	14	10	6
	Lower	100	70	53	44	39	31	20	15	10	8	5	3	2
	Median	100	80	62.5	55	49.5	41	30	23.5	17.5	13	9.5	6.5	4

### 2.3. Rutting test method

#### 2.3.1. Rutting test condition.

Asphalt mixture is typical viscoelastic materials, and its deformation resistance is affected by temperature and load significantly. To evaluate the effects of temperature and vehicle load on deformation resistance of asphalt mixture, the index of deformation resistance, mainly dynamic stability and rutting deformation, were tested under different conditions of temperature and vehicle load. According to the climate characteristics and temperature of the pavement structure, the test temperatures (T) are set as 20^o^C, 30^o^C, 40^o^C, 50^o^C, 60^o^C, and 70^o^C in this study. This temperature region encompasses the entire process of asphalt pavement transitioning from elastic-dominated state to viscoelastic-dominated state, and this range covers pavement conditions from normal temperatures to extreme heat in China. Based on the actual stress exerted by wheels on the road surface and fully considering the high proportion of heavy-duty vehicles in China’s transportation, the test loads (P) were set as 0.5 MPa, 0.7 MPa, 0.9 MPa, and 1.1 MPa, respectively.

#### 2.3.2. Preparation of rutting plate specimen.

**Fig 2:**
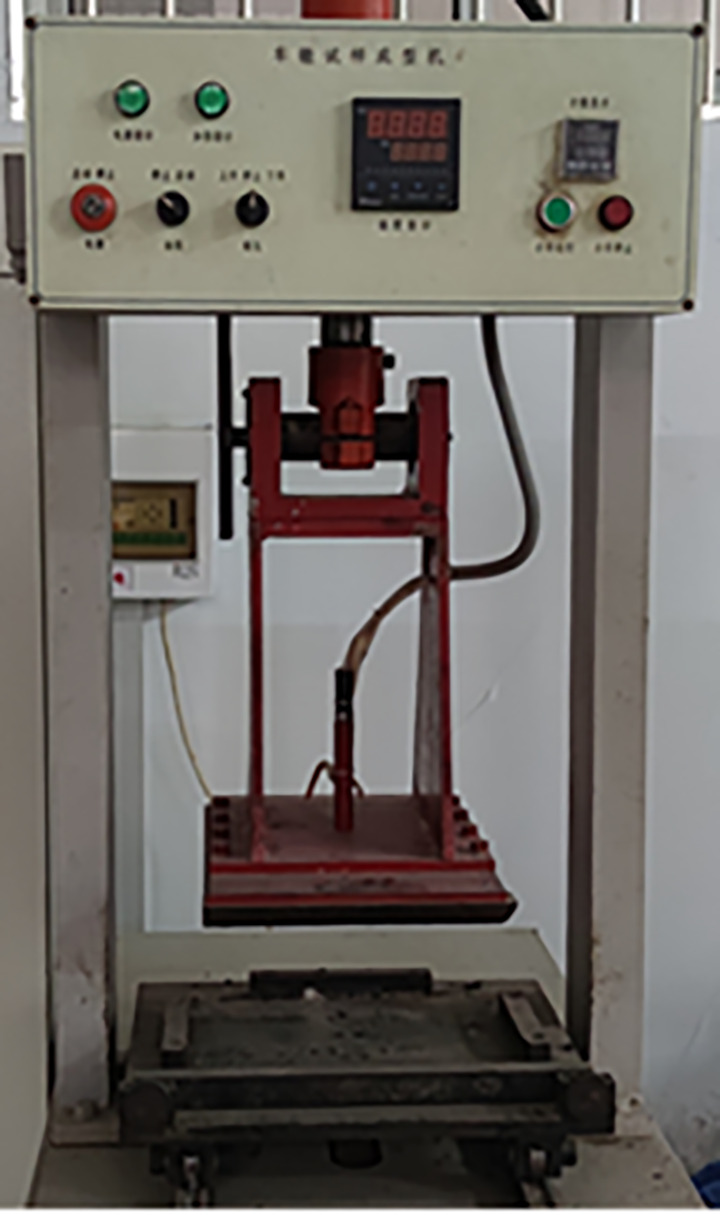
Rutting specimen molding machine.

The rutting plate specimens of AC-13, AC-20 and ATB-30 were formed in laboratory used the rutting specimen molding machine (shown in Fig 2), and the gradations used in these tests are shown in Fig 1. In the tests, AC-13, AC-20 and ATB-30 were used with target voids of 4.0%, 4.2%, and 4.4%respectively. Marshall test method was utilized to determine the optimum asphalt-aggregate ratio for AC-13, AC-20, and ATB-30 which were 4.8%, 4.2%, and 3.6% respectively. The rutting plate specimens, 300 mm wide by 300 mm long by 50 mm thick, were prepared for AC-13 and AC-20, and 300 mm wide by 300 mm long by 80 mm thick for ATB-30, which reference to T0703-2011 of the Chinese specification of JTG 3410−2025 [36]. In the rutting tests, the mixing temperature of the asphalt mixture was 140^o^C to 160^o^C and the compaction temperature was 120^o^C to 150^o^C. Before formal compaction of the rutting specimen, its density should be measured after pressure test. After the specimens are molded, the rolling direction is marked with chalk, and the specimens are cooled naturally at room temperature for at least 12 hours before it is demolded. In the rutting test, three parallel specimens were performed in each test, and the average value of the three test results was taken as the test results. The variation coefficient of the test results was controlled within 20%.

#### 2.3.3. Rutting test.

**Fig 3:**
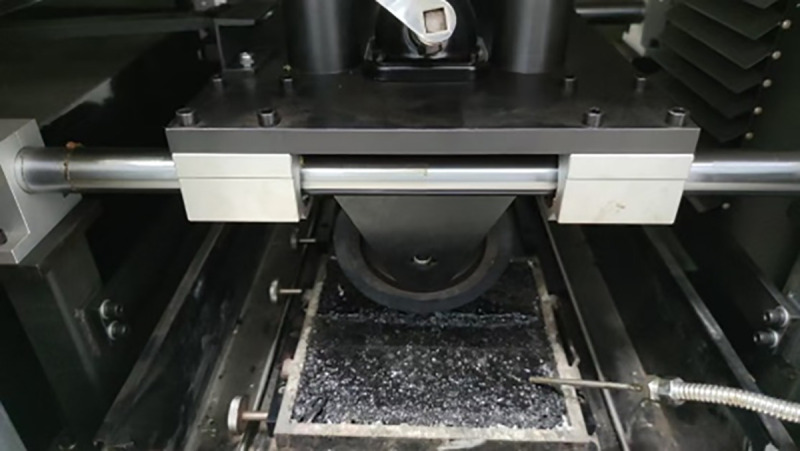
Rutting test.


The rutting test is carried out with reference to T0719-2025 of the Chinese specification of JTG 3410−2025 [36], and the loading rate is 42 times/min. The wheel rutting tester and rutting test were shown in Fig 3. The dynamic stability calculated using Eq. (1).

$$DS=C_{1}\times \frac{t_{2}-t_{1}}{d_{2}-d_{1}}$$ (1)

Where *DS* is the dynamic stability, times/mm; *t*_1_ and *t*_2_ are the loading times, which is 1890 times and 2520 times, respectively; *d*_1_ and *d*_*2*_ are the rutting deformation at *t*_1_ and *t*_2_, mm; *C*_1_ is the coefficient of the specimen width, for a standard specimen with a width of 300 mm, its value is 1.0.

## 3. Results and discussion

### 3.1. Results of rutting tests

Table 5 shows the test results of dynamic stability (*DS*) and rutting deformation at load time of 2520 (*RD*_2520_) and 12600 (*RD*_12600_) for AC-13, AC-20 and ATB-30 with variable temperature and loads. It could be noted that *RD*_2520_, *RD*_12600_ and *DS* were temperature and load sensitive.

**Table 5:** Test results and rutting deformation indicators.

Mixture	P (MPa)	T (°C)	*RD*_2520_ (mm)	*RD*_12600_ (mm)	*DS* (times/mm)	*CSI* (times/mm^2^)	*D*	*1/E*	*A*	*C*	*A/C*
AC-13	0.5	70	6.08	10.26	776	145	1.669	545	36.0	0.199	181
		60	2.87	3.48	2369	878	1.077	1290	15.1	0.171	88
		50	2.17	2.58	5526	2646	0.936	1827	10.9	0.159	69
		40	1.54	1.76	6843	4905	0.545	2378	8.2	0.176	47
		30	1.11	1.28	11518	10841	0.427	3241	6.1	0.174	35
		20	0.79	0.94	14533	20234	0.260	4373	4.5	0.185	24
	0.7	70	8.09	12.64	672	96	2.043	407	48.9	0.205	238
		60	4.06	4.93	1981	520	1.792	1063	18.7	0.150	125
		50	2.84	3.31	4627	1699	1.397	1652	12.6	0.138	91
		40	1.75	2.10	6372	3886	0.646	2106	9.2	0.172	53
		30	1.26	1.47	10090	8508	0.488	3031	6.4	0.166	38
		20	0.86	1.14	13777	17556	0.307	4270	4.6	0.176	26
	0.9	70	8.82	14.31	597	78	2.346	378	52.0	0.200	260
		60	4.30	5.40	1721	433	1.646	899	21.4	0.166	130
		50	3.12	3.80	3210	1084	1.638	1717	12.5	0.124	101
		40	2.04	2.47	5546	2876	0.951	2230	9.2	0.144	64
		30	1.38	1.63	9121	6940	0.628	3134	6.5	0.151	43
		20	0.93	1.19	12857	15014	0.315	3806	5.1	0.181	28
	1.1	70	9.75	15.82	551	65	2.621	336	58.8	0.201	292
		60	5.07	7.14	1549	340	1.960	788	24.6	0.163	151
		50	3.44	4.41	2745	854	1.772	1523	13.9	0.126	110
		40	2.21	2.75	4930	2372	1.048	2104	9.7	0.140	69
		30	1.59	1.81	8186	5349	0.782	2907	7.2	0.141	51
		20	0.98	1.29	11560	12827	0.381	4034	4.8	0.162	29
AC-20	0.5	70	4.97	8.21	813	190	1.317	670	29.4	0.201	146
		60	2.18	2.97	3651	1847	0.769	1681	11.5	0.175	66
		50	1.62	2.11	5700	3831	0.622	2372	8.2	0.167	49
		40	0.98	1.35	9460	10653	0.339	3691	5.2	0.177	29
		30	0.56	0.73	13255	25598	0.187	6127	3.2	0.184	17
		20	0.21	0.30	15867	32865	0.046	13747	1.5	0.219	7
	0.7	70	7.04	11.11	773	127	1.781	468	42.5	0.205	207
		60	2.90	4.13	2933	1127	1.208	1462	13.3	0.153	87
		50	2.16	2.79	5132	2567	0.969	2065	9.7	0.146	66
		40	1.22	1.62	8397	7522	0.452	3082	6.3	0.171	37
		30	0.71	0.90	12054	18472	0.257	5199	3.7	0.173	21
		20	0.35	0.52	14376	46497	0.122	10462	1.8	0.173	10
	0.9	70	7.50	12.18	750	116	2.011	444	44.3	0.200	222
		60	3.20	4.47	2243	782	1.086	1147	16.7	0.177	94
		50	2.14	3.17	4188	2200	0.865	1936	10.0	0.157	64
		40	1.44	1.96	7067	5356	0.662	3155	6.4	0.143	45
		30	0.76	1.05	11440	16554	0.275	4861	4.0	0.172	23
		20	0.48	0.76	13743	32526	0.151	7036	2.8	0.189	15
	1.1	70	8.46	13.61	642	88	2.267	388	51.0	0.201	253
		60	3.53	5.69	2197	717	1.326	1106	17.4	0.166	105
		50	2.47	3.64	3684	1674	1.015	1690	11.6	0.157	74
		40	1.65	2.32	6180	4126	0.759	2739	7.3	0.143	51
		30	0.97	1.35	9772	11120	0.364	3960	4.8	0.166	29
		20	0.59	0.81	12448	23415	0.160	5432	3.6	0.201	18
ATB-30	0.5	70	3.56	6.64	1015	387	0.214	773	35.9	0.289	124
		60	1.70	2.47	3352	2320	0.272	1671	12.7	0.236	54
		50	1.38	1.88	5351	4433	0.397	2391	8.2	0.196	42
		40	0.89	1.24	6470	8902	0.218	3612	5.6	0.210	27
		30	0.46	0.61	8430	22428	0.068	6022	3.6	0.240	15
		20	0.24	0.36	10702	31376	0.011	10669	2.9	0.304	10
	0.7	70	4.52	7.59	1022	285	2.819	322	34.4	0.245	140
		60	1.95	2.73	3171	1869	0.523	1671	11.8	0.201	59
		50	1.48	1.99	3925	3057	0.498	2391	8.1	0.181	45
		40	0.91	1.26	8832	10937	0.631	4563	5.5	0.202	27
		30	0.48	0.63	11776	27847	0.093	6022	3.4	0.226	15
		20	0.27	0.39	19022	38895	0.036	10661	2.5	0.269	9
	0.9	70	5.12	8.19	985	236	3.423	670	30.4	0.205	148
		60	2.08	2.78	3989	2154	0.963	2134	9.5	0.145	65
		50	1.21	1.60	8832	8158	0.434	3068	6.3	0.174	36
		40	0.69	0.89	9511	15428	0.240	5168	3.7	0.178	21
		30	0.34	0.50	16488	28310	0.105	10304	1.9	0.187	10
		20	0.18	0.28	82461	303710	0.031	17221	1.2	0.231	5
	1.1	70	5.10	9.50	629	173	0.652	599	44.2	0.270	163
		60	3.15	4.38	1781	665	0.985	1131	17.0	0.185	92
		50	2.02	3.07	3180	1833	0.777	1962	9.8	0.163	60
		40	1.31	1.87	6547	5600	0.577	3256	6.1	0.151	40
		30	0.67	0.95	7949	13766	0.181	4835	4.1	0.202	20
		20	0.39	0.66	13092	38870	0.057	6984	3.2	0.246	13

To illustrate the progression of rutting deformation, plots the variation of rutting depth (RD) with loading cycles under a load of 0.7 MPa. To characterize the effect of temperature and load on the rutting deformation visually, Fig 7 illustrates the 3D surface plot of *RD*_12600_ of the mixtures versus temperature (T) and load (P).

**Fig 4:**
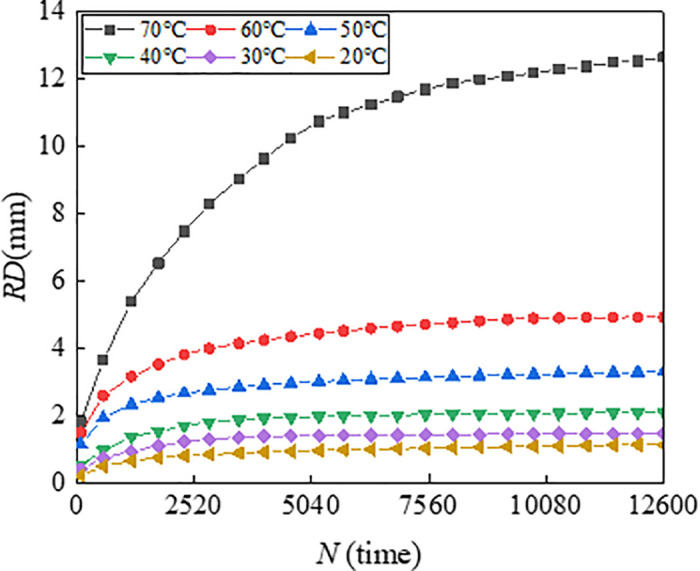
Variation of *RD* with *N* for asphalt mixture of AC-13.

**Fig 5:**
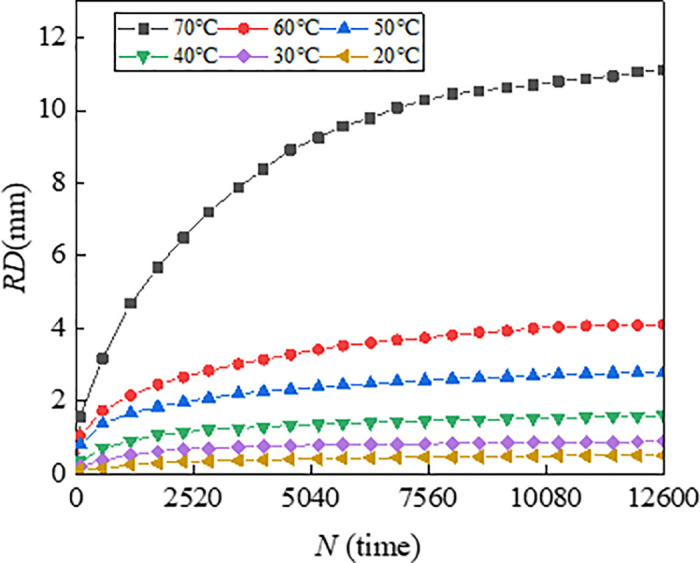
Variation of *RD* with *N* for asphalt mixture of AC-20.

**Fig 6:**
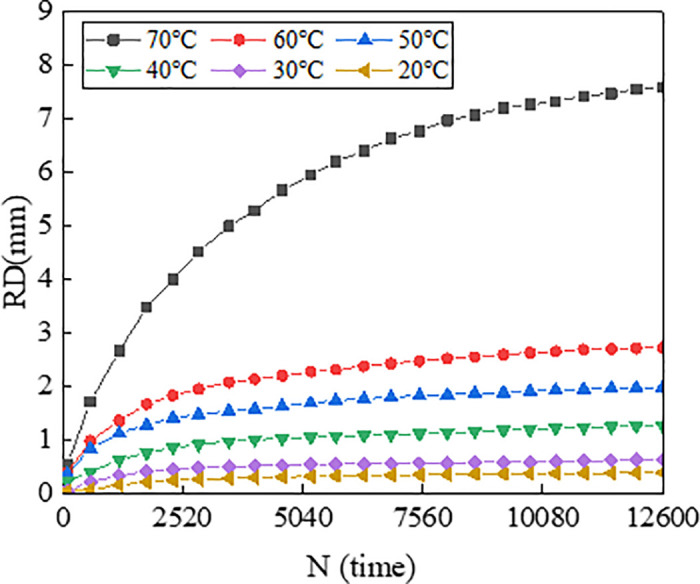
Variation of *RD* with *N* for asphalt mixture of ATB-30.

**Fig 7:**
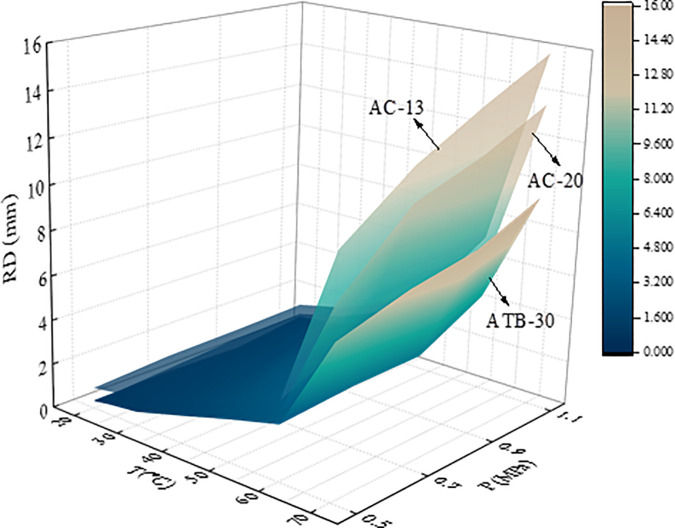
3D surface plot of *RD*_12600_ and *T*-*P.*

From Fig 7, the rutting deformation development at different temperatures for each load are very comparable with the increase of loading cycles. The test results are consistent with research results of Zhou et al., which indicate that rutting deformation all tend to increase with increases in load and temperature [38]. It is found that the rutting growth rate of AC-13 is higher than that of AC-20 and ATB-30 during the initial stage of loading and reaches the steady state earlier. This is because the size of the coarse aggregate of AC-13 is smaller compared to AC-20 and ATB-30, and the skeleton formed by the coarse aggregate is poor, which resulting in weak rutting resistance, and the fine aggregate and asphalt can be pressed into the void easily under load. Correspondingly, the coarse aggregate of ATB-30 forms a skeleton with high strength, which makes it difficult for the coarse aggregate to be displaced due to the friction and bonding force under the load, and the skeleton damage degree is low, which makes it difficult for the fine aggregate to enter the void.

Fig 7 shows that the 3D surface plot of AC-13 is above of AC-20 and ATB-30, which indicating that the *RD* of ATB-30 is smaller than that of AC-13 and AC-20 under the same temperature and loading conditions. In other words, the high temperature performance of ATB-30 is better than that of AC-13 and AC-20. Take AC-13 as example, the rutting deformation under high temperature and heavy loads is about 15 times higher than that under low temperature and light loads (15.3 mm for 70°C and 1.1MPa, 1.01 mm for 20°C and 0.5MPa). Therefore, heavy load and high temperature have a significant effect on rutting deformation.

### 3.2. Coupling effect of temperature and load

The sensitivity of temperature and wheel load to the dynamic stability and rutting deformation at 0.05 level of significance was investigated by analysis of variance (ANOVA). The results of ANOVA are presented in Table 6. In Table 6, SS represent sum of squares and MS is mean sum of squares.

**Table 6:** ANOVA results for DS and RD of asphalt mixtures.

Indicator	Mixture	Factor	SS	MS	F-value	P-value
DS	AC-13	T (°C)	4.59 × 10^8^	9.18 × 10^7^	290	2.26 × 10^-14^
		P (MPa)	1.38 × 10^7^	4.59 × 10^6^	15	1.05 × 10^−4^
	AC-20	T (°C)	5.40 × 10^8^	1.08 × 10^8^	317	1.18 × 10^-14^
		P (MPa)	1.74 × 10^7^	5.81 × 10^6^	17	4.23 × 10^−5^
	ATB-30	T (°C)	6.35 × 10^9^	1.59 × 10^9^	53	1.63 × 10^−7^
		P (MPa)	2.36 × 10^9^	7.87 × 10^8^	26	1.51 × 10^−5^
RD	AC-13	T (°C)	413	83	106	3.60 × 10^-11^
		P (MPa)	15	5	6	5.32 × 10^−3^
	AC-20	T (°C)	315	63	100	5.73 × 10^-11^
		P (MPa)	12	4	6	5.23 × 10^−3^
	ATB-30	T (°C)	2.5	0.6	62.5	6.19 × 10^−8^
		P (MPa)	0.8	0.3	25.6	1.68 × 10^−5^

From Table 6, it can be seen that the F-value corresponding to temperature is greater than that corresponding to load for the three asphalt mixtures, that is, the sensitivity of the dynamic stability and rutting deformation to temperature is higher than that to load. The P-value corresponding to temperature and load are less than 0.05 for the three asphalt mixtures, illustrating that the dynamic stability and rutting deformation is more sensitive to temperature than load at 0.05 significance level.

### 3.3. Correlation analysis of RD_2520_ and RD_12600_

**Fig 8:**
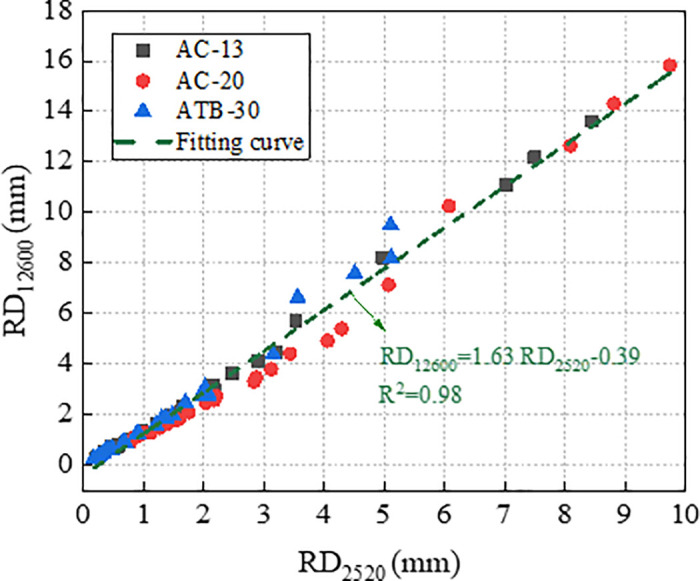
Correlation of RD_2520_ and RD_12600_.

The correlation of RD_2520_ and RD_12600_ for the three asphalt mixture is shown in Fig 8.

Fig 8 shows that RD_2520_ and RD_12600_ are highly linearly correlated, and the correlation coefficient (R^2^) exceed 0.98. Therefore, the RD_2520_ can be used to predict RD_12600_.

### 3.4. Rutting deformation growth pattern

The whole process of rutting deformation development could be divided into three phases. The primary phase of rutting deformation is densification of the mixture, in which the strain rate decreases swiftly with the increase of loading cycles. The secondary phase is shear flow of the mixture, in which the strain rate is nearly constant. The tertiary phase is shear failure of the mixture, in which the strain rate increases dramatically with the increase of loading cycles [39,40]. However, taking into account the equipment and efficiency of the rutting test, it is difficult to reach the tertiary phase in laboratory test. And even with five hours of rutting tests, the rutting deformation still cannot reach the tertiary phase. Therefore, the growth pattern of rutting deformation in the first two phases were mainly investigated in the laboratory test.

**Fig 9:**
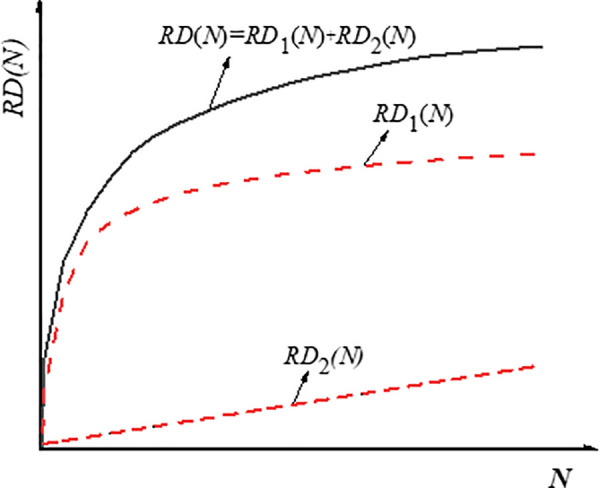
Two phases of rutting deformation development.


Gladkikh et al. presented a two phases analytical model which describes the primary and secondary phases of rutting deformation [41]. Fig 9 shows the two phases of rutting deformation, the primary phases can be descried by exponential function, and the second phases can be described by linear function. The model can be described by Eq. (2).

$$RD(N)=RD_{1}(N)+RD_{2}(N)=D\frac{1-e^{-bN}}{1+e^{-bN}}+E\cdot N$$ (2)

As shown in Eq. (2), it can be easily deduced that the limit of the first term is equal to a constant value, as shown in Eq. (3). The constant value *D* was defined as densification coefficient.


$$\underset{N\to \infty }{\lim}RD_{1}(N)=\underset{N\to \infty }{\lim}D\frac{1-e^{-bN}}{1+e^{-bN}}=D$$ (3)

The parameter *E* in the second term of Eq. (2) is the slope of the line, which is defined as the rate of plastic flow, and the index ‘1/*E*’ was labelled as shear index.

The rutting deformation development also can be simulated by another model, which is shown by Eq. (4) [42].


$$RD(N)=Ae^{-{\left(B/N\right)}^{C}}$$ (4)

To eliminate the variability of parameter values while fitting rutting curves, B. Javilla etc. fixed the parameter *B* as 50000 empirically through experimental research [43]. The modified model is shown by Eq. (5).


$$RD(N)=Ae^{-{\left(50000/N\right)}^{C}}$$ (5)

Parameter *A* and *C* can be determined by means of nonlinear regression, the index ‘*A/C*’ was labelled as characteristic parameters of rutting deformation.

### 3.5 Deformation resistance indicators

The deformation resistance indicators of AC-13, AC-20 and ATB-30 can be determined by means of nonlinear regression. Actually, the densification coefficient (*D*) and shear index (*1/E*) can be determined by Eq. (2), and A and *C* can be determined by Eq. (5), all the parameters are shown in Table 4.

#### 3.5.1. Dynamic stability and complex stability index.

The dynamic stability is an effective indicator to evaluate high temperature performance of asphalt mixture. Base on the dynamic stability, Du and Dai proposed complex stability index (*CSI*) to evaluate the rutting performance of asphalt mixture, and the results showed that *CSI* was a better indicator of asphalt mixture[20]. The *CSI* was determined by Eq. (6). And the results of CSI were shown in Table 4.


$$CSI\;=\frac{t_{2}-t_{1}}{d_{1}(d_{2}-d_{1})}\times C_{1}=\frac{DS}{d_{1}}$$ (6)

**Fig 10:**
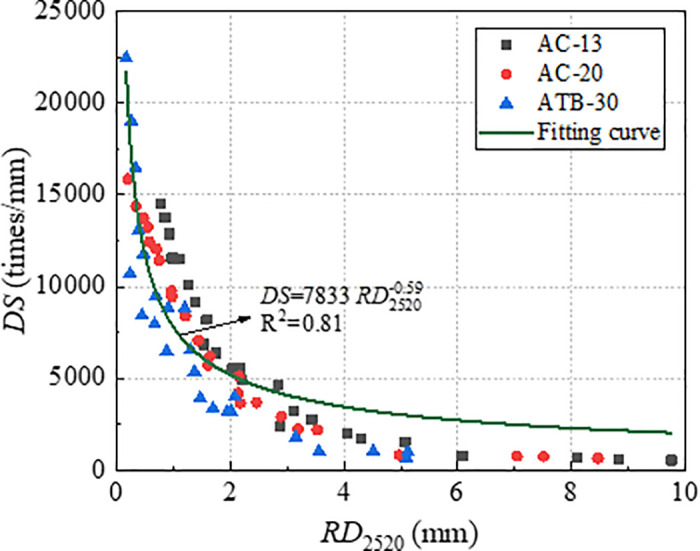
Correlation of RD_2520_ with *DS.*

**Fig 11:**
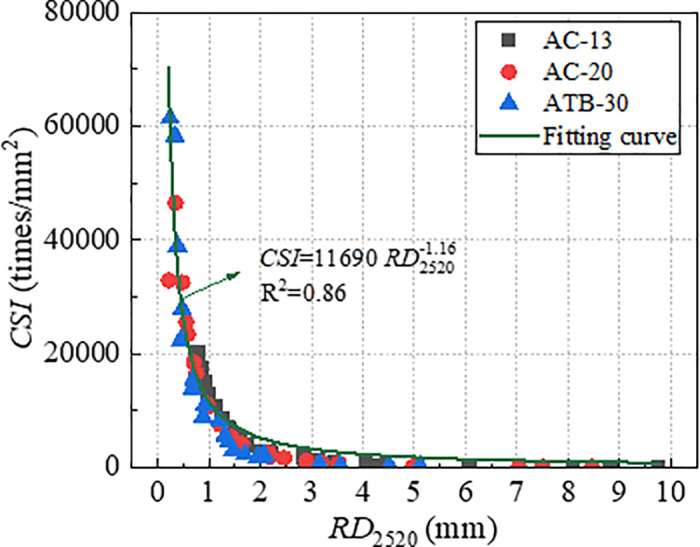
Correlation of RD_2520_ with *CSI.*

The correlations of *RD*_2520_ with *DS* and *CSI*, are shown in Figs 10 and 11. It could be seen that *RD*_2520_ exhibits a nonlinear relationship with both *DS* and *CSI*, and the correlation coefficient between *CSI* and *RD*_2520_ is higher than that between *DS* and *RD*_2520_. This was in agreement with the study by Du and Dai, where the authors pointed out that the index *CSI* was more effective than index *DS* in evaluating the deformation resistance of asphalt mixture.

**Fig 12:**
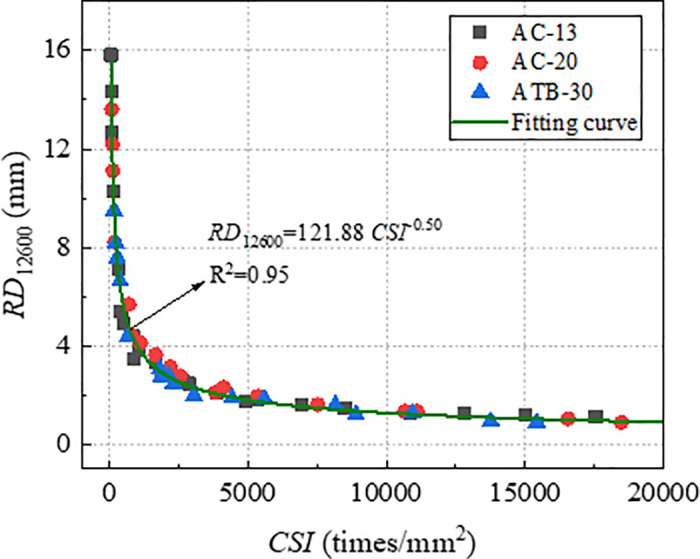
Correlation of *RD*_12600_ and *CSI.*

To validate the effectiveness of index *CSI* in characterizing the long-term deformation of rutting, the index *CSI* which were obtained by *RD*_2520_ were used to establish the relationships with *RD*_12600_. The relationship curve between *RD*_12600_ and *CSI* was plotted, as shown in Fig 12. As evident from Fig 12, *RD*_12600_ and *CSI* exhibit a nonlinear correlation with a high correlation coefficient of 0.95, which was significantly stronger than the correlation coefficient *RD*_2520_ and *CSI.* This demonstrates that index *CSI* can characterize long-term rutting deformation effectively. Therefore, *CSI* could be considered as an efficient indicator to evaluate rutting performance of asphalt mixture.

#### 3.5.2. Densification coefficient and shear index.

**Fig 13:**
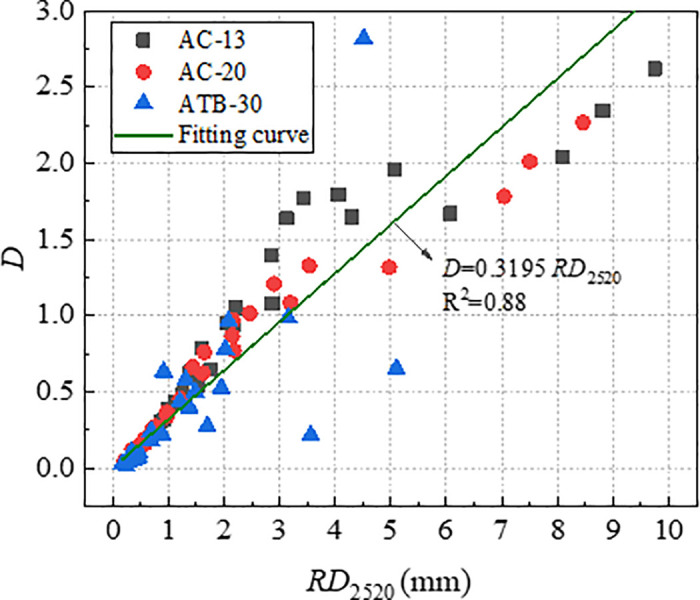
Correlation of RD_2520_ with *D.*

**Fig 14:**
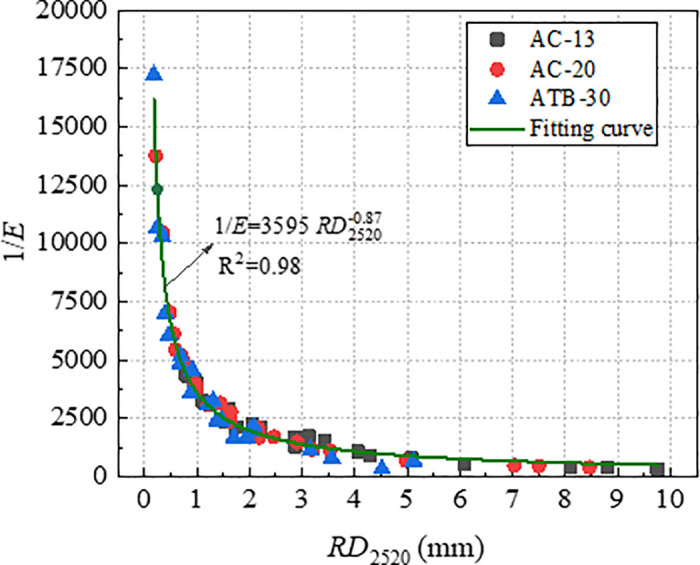
Correlation of RD_2520_ with *1/E.*

According to the data in Table 4, plot the relationship curves between *RD*_2520_ and *D*, as well as between *RD*_2520_ and *1/E*, as shown in Figs 13 and 14. As shown in Figs 13 and 14, the parameters (*D* and *1*/*E*) were significantly correlated with *RD*_2520_. The index *1*/*E* had a stronger correlation coefficient of 0.98 than index *D*. This was reasonable because rutting is attributed primarily to the shear flow characteristics of asphalt mixture other than initial densification.

**Fig 15:**
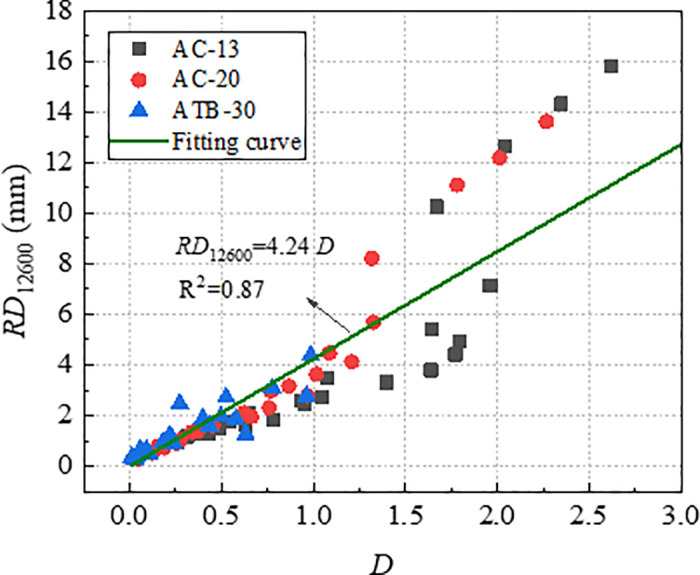
Relationships between *D* and *RD*_12600_.

**Fig 16:**
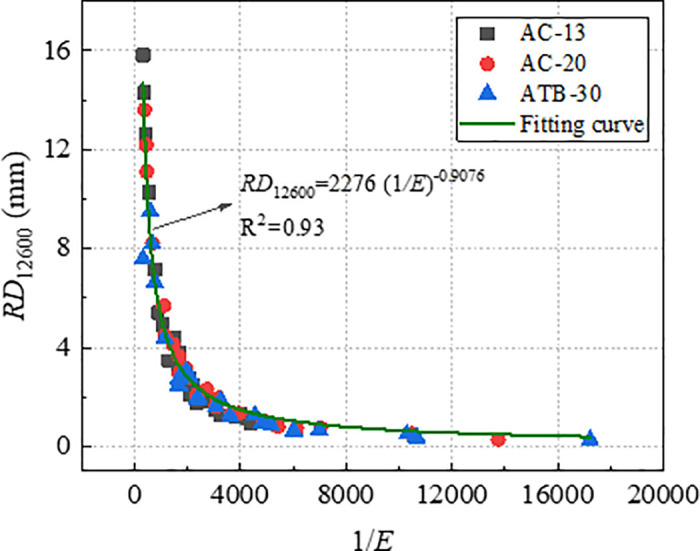
Relationships between 1/*E* and *RD*_12600_.

With a view to verifying the effectiveness of index *D* and *1*/*E* in characterizing long-term rutting deformation, the index *D* and *1*/*E* which were obtained by *RD*_2520_ were used to establish the relationships with *RD*_12600_. The relationship curve between *RD*_12600_ with *D* and *1*/*E* were plotted, as shown in Figs 15 and 16. As evident from Figs 15 and 16, both *RD*_12600_ and *D*, *RD*_12600_ and *1*/*E* exhibit high correlation coefficient. This indicates that index *D* and *1*/*E* can effectively characterize long-term deformation of rutting. Therefore, both *D* and *1*/*E* could be considered as efficient indicators to evaluate rutting performance of asphalt mixture.

#### 3.5.3 Characteristic parameters of rutting deformation.

**Fig 17:**
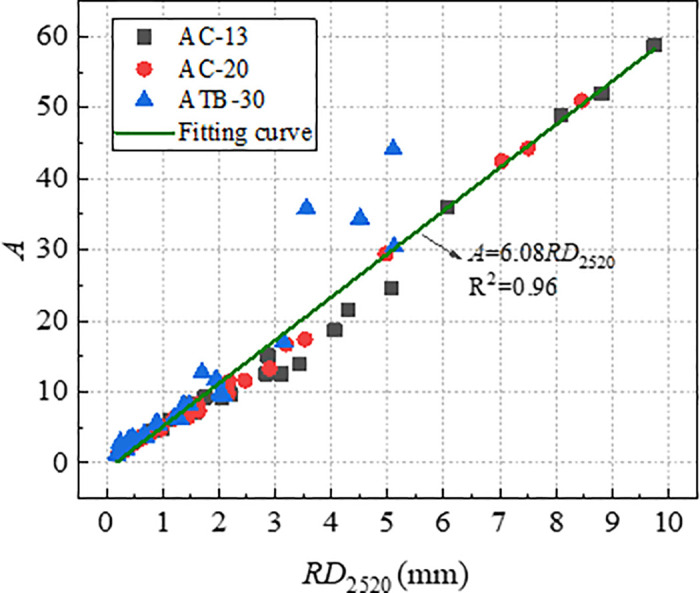
Correlation of *RD*_2520_ with *A.*

**Fig 18:**
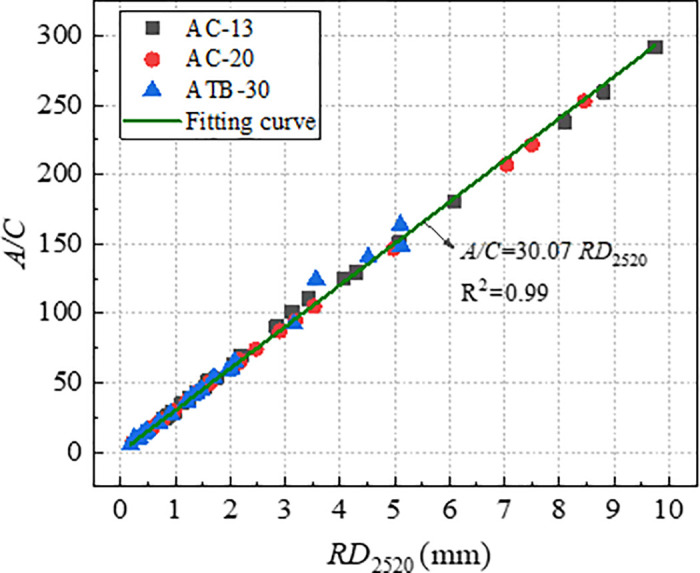
Correlation of *RD*_2520_ with *A/C.*

The index *A* and *C* was determined based on the Eq. (5) by means of non-linear regression, and the index *A/C* was labelled as characteristic parameters of rutting deformation. Figs 17 and 18 shows the correlation of *RD*_2520_ with *A* and *A/C*. From Figs 17 and 18, the *RD*_2520_ is linearly and positively correlated with both *A* and *A/C*, and the index *A/C* was more reliable rutting indicator than index *A*. Compare to the index *DS* and *CSI*, the index *A/C* had the strongest correlation coefficient.

**Fig 19:**
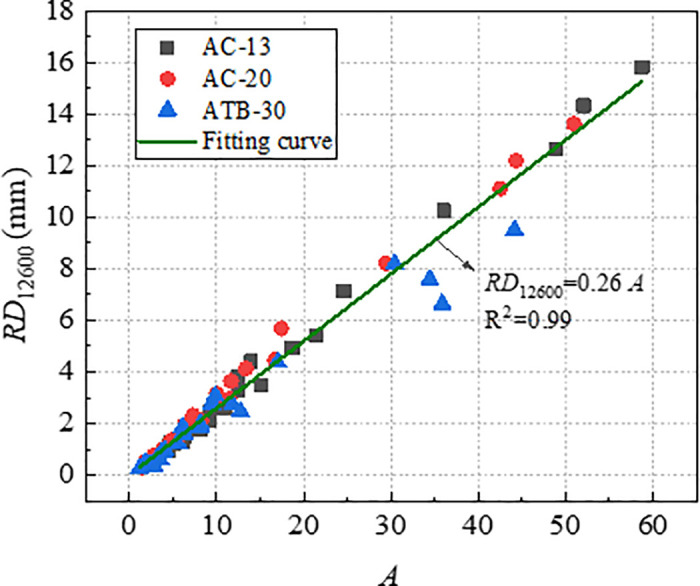
Correlation of *A* with *RD*_12600_.

**Fig 20:**
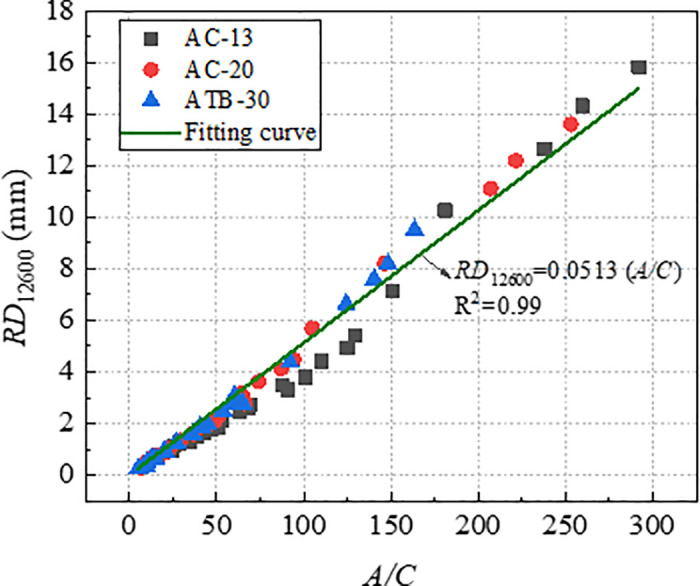
Correlation of *A/C* with *RD*_12600_.

To verify the effectiveness of index *A* and *A/C* in characterizing long-term rutting deformation, the index *A* and *A/C* which were obtained by *RD*_2520_ were used to establish the relationships with *RD*_12600_. Figs 19 and 20 shows the correlation of *RD*_12600_ with *A* and *A/C*, and the correlation coefficients are as high as 0.99, which indicted that *A* and *A/C* could be used for predicting the long-term deformation of rutting for asphalt mixtures. Therefore, *A* and *A/C* could be considered as efficient indicators to evaluate rutting performance of asphalt mixtures.

#### 3.5.4. Comparison of rutting performance indicators.

**Fig 21:**
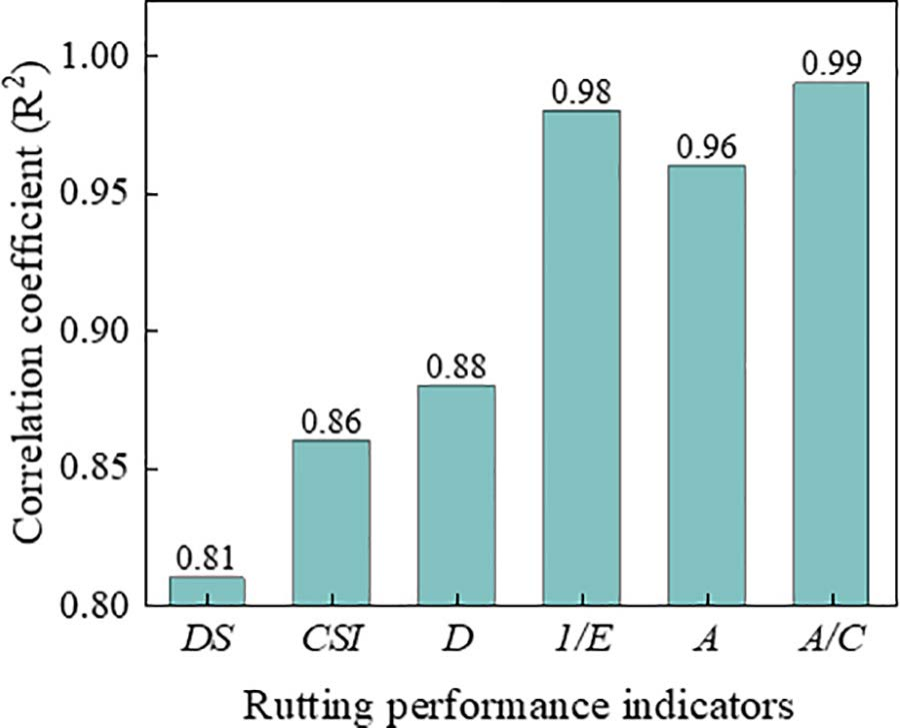
Comparison of correlation coefficient for *RD*_2520_.

**Fig 22:**
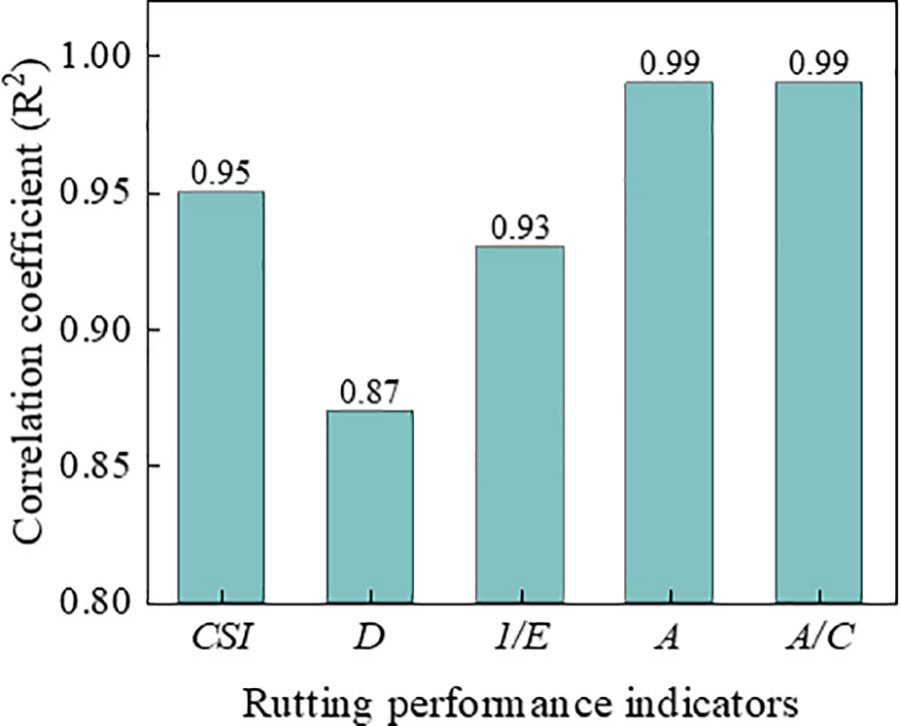
Comparison of correlation coefficient for *RD*_12600_.

Figs 21 and 22 shows the comparison of correlation coefficients between various indicators and rutting deformation (*RD*_2520_ and *RD*_12600_). The correlation coefficients with *RD*_12600_ for all indicators except indicator of *D* were exceed 0.90, which indicating that these indicators can characterize the deformation resistance of asphalt mixtures effectively. Furthermore, the relationship between the indicators and *RD*_12600_ also can be used to predict the long-term deformation of rutting in laboratory test. Since the index *A* and *A/C* were both exhibit high correlation with rutting deformation, then it is sufficient to select the index *A/C* for practical application.

### 3.6. Validation of indicators

In order to provide further verification of the reliability and effectiveness of the proposed indicators of deformation resistance, the rutting specimens were prepared in laboratory with the gradations which were shown in Fig 1. Multiple rutting tests were conducted under the same test temperature and loading conditions. The test results were shown in Table 7.

**Table 7:** Test resultsof the validation test.

Mixture	Test No.	P (MPa)	T (°C)	*RD*_2520_ (mm)	*CSI* (times/mm^2^)	*D*	*1/E*	*A*	*C*	*A/C*
AC-13	1	0.7	60	3.907	1163	1.532	901	19.5	0.161	121
	2			3.741	2816	1.896	1052	17.2	0.132	131
	3			3.496	3169	1.792	1141	15.4	0.124	125
	1	0.9	40	2.047	3186	0.740	1652	11.4	0.181	63
	2			1.988	3950	0.979	2077	8.8	0.130	67
	3			1.841	3118	0.845	2171	8.2	0.139	59
AC-20	1	0.7	60	2.786	1301	1.025	1260	13.2	0.158	84
	2			2.563	2576	1.241	1537	11.9	0.140	85
	3			2.696	2605	1.353	1474	12.5	0.135	93
	1	0.9	40	1.493	3640	0.590	2450	7.5	0.163	46
	2			1.370	5279	0.627	2856	5.9	0.134	44
	3			1.456	11550	0.750	2923	6.4	0.126	51
ATB-30	1	0.7	60	0.733	14163	0.186	3995	4.7	0.202	23
	2			0.835	17036	0.234	3406	5.4	0.198	28
	3			0.750	26615	0.259	4084	4.6	0.184	25
	1	0.9	40	0.537	33329	0.081	4617	4.3	0.237	18
	2			0.594	15829	0.168	4717	3.6	0.187	19
	3			0.566	53651	0.177	5186	3.6	0.194	19

**Fig 23:**
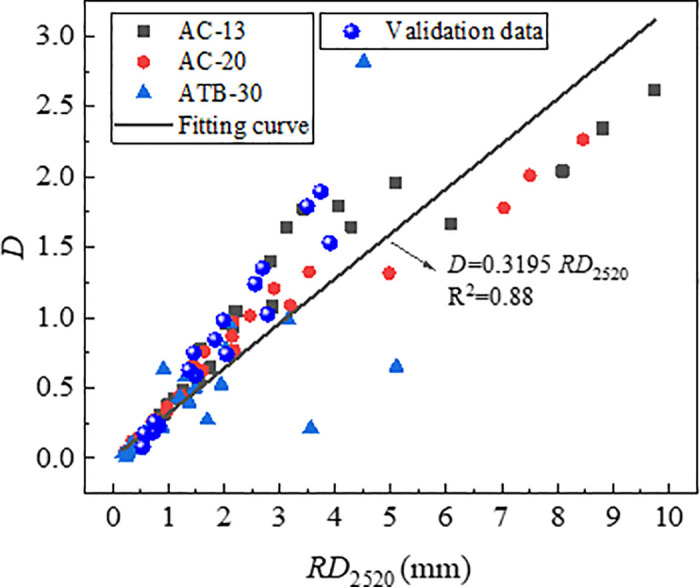
Test results verification of *D.*

**Fig 24:**
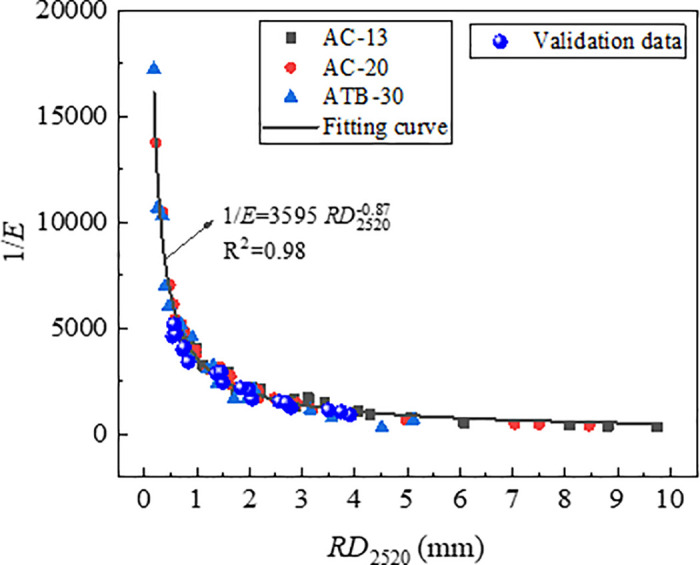
Test results verification of 1/*E.*

**Fig 25:**
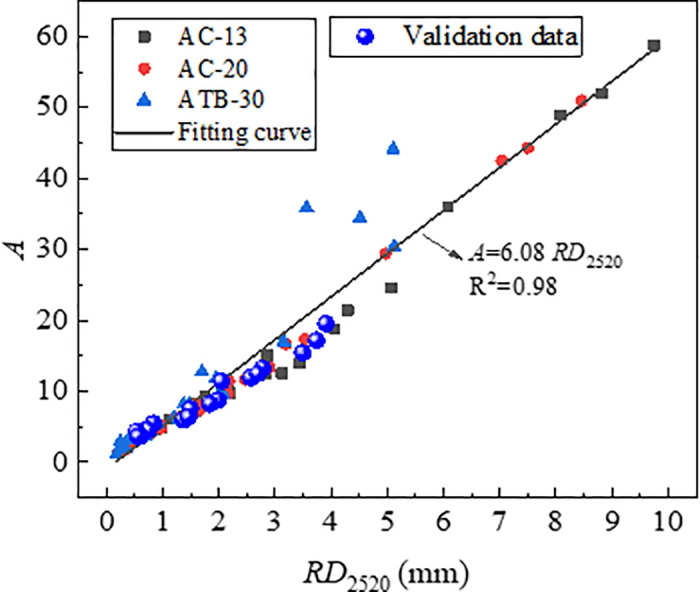
Test results verification of *A.*

**Fig 26:**
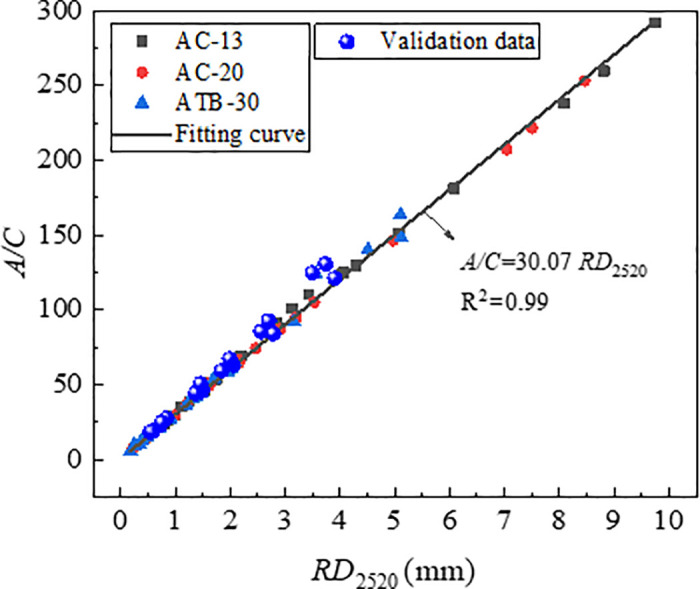
Test results verification of *A/C.*

**Fig 27:**
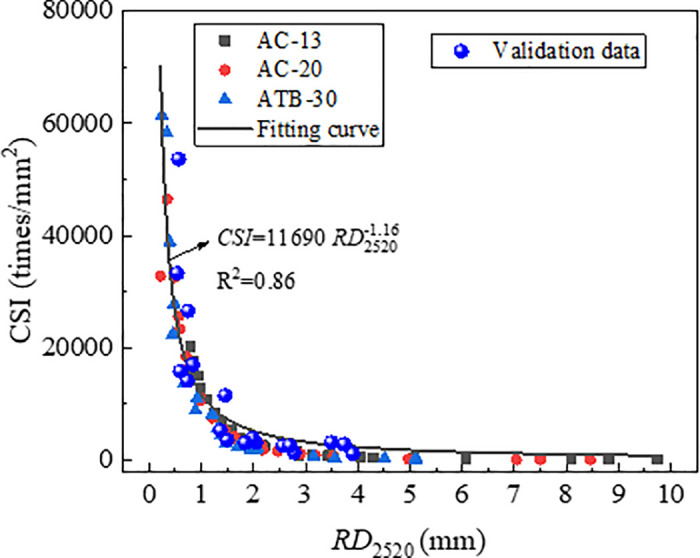
Test results verification of *CSI.*

The test data from Table 7 were plotted on the relationship curves between each indicator and RD_2520_ which were established previously. The results are presented in Figs 23–27. As is shown in Figs 23–27, all the test data are distributed near the respective curves, which demonstrates that these indicators are effective in characterizing rutting deformation.

## 4. Conclusion and outlook

The deformation resistance of AC-13, AC-20 and ATB-30 asphalt mixtures with variable temperature and loads were investigated in this study, and the rutting performance indicators were proposed based on the rutting test results and rutting deformation growth pattern, then the following conclusions are derived.

(1) The deformation resistance of AC-13, AC-20 and ATB-30 asphalt mixtures were all affected by temperature and load significantly, and the deformation resistance is more sensitive to temperature than load at 0.05 significance level.
(2) All the rutting performance indicators proposed in this study, namely *CSI*, *D*, *1/E*, and *A/C* were significantly correlation with rutting deformation.
(3) The proposed deformation resistance indicators can accurately predict the permanent rutting deformation in laboratory rutting tests.

This study proposes the deformation resistance indicators for conventional asphalt mixtures. However, with the application of new materials such as high modulus asphalt, rubber modified asphalt, warm mix additives, and fibers, as well as novel structural layers like large-sized permeable and high-elasticity layers, taking into account the influence of multiple factors, such as humidity and aging effect, it is necessary to systematically validate and adjust the sensitivity of the proposed indicators to these new materials. Multiple materials will be employed to validate the reliability of these indicators and establish corresponding standards based on engineering practice in future research. Furthermore, the engineering applications of these indicators will also be incorporated into future research.

## Supporting information

S1 File Date. This file includes all the test data of the asphalt and asphalt mixtures.
(DOCX)
