# Supplementary material for: Laboratory study on the deformation resistance indicators of asphalt mixture based on the rutting deformation growth model
Source: PLoS One. 2026 Feb 12;21(2):e0340720. doi: 10.1371/journal.pone.0340720 (PMC12900368; doi:10.1371/journal.pone.0340720)
Supplement: S1 File — This file includes all the test data of the asphalt and asphalt mixtures. (DOCX) [file pone.0340720.s001.docx]

**Table 1**

| Mixture | Gradation | Passage rate (%) through the following sieves (mm) | | | | | | | | | | | | |
| --- | --- | --- | --- | --- | --- | --- | --- | --- | --- | --- | --- | --- | --- | --- |
|  |  | 31.5 | 26.5 | 19 | 16 | 13.2 | 9.5 | 4.75 | 2.36 | 1.18 | 0.6 | 0.3 | 0.15 | 0.075 |
| AC-13 | Upper | - | - | - | 100 | 100 | 80 | 50 | 40 | 28 | 20 | 15 | 11 | 8 |
|  | Lower | - | - | - | 100 | 90 | 65 | 40 | 30 | 18 | 11 | 7 | 5 | 4 |
|  | Median | - | - | - | 100 | 95 | 72.5 | 45 | 35 | 23 | 15.5 | 11 | 8 | 6 |
| AC-20 | Upper | - | 100 | 100 | 85 | 70 | 55 | 38 | 28 | 20 | 16 | 13 | 10 | 7 |
|  | Lower | - | 100 | 90 | 70 | 55 | 45 | 28 | 18 | 12 | 8 | 5 | 4 | 3 |
|  | Median | - | 100 | 95 | 77.5 | 62.5 | 50 | 33 | 23 | 16 | 12 | 9 | 7 | 5 |
| ATB-30 | Upper | 100 | 90 | 72 | 66 | 60 | 51 | 40 | 32 | 25 | 18 | 14 | 10 | 6 |
|  | Lower | 100 | 70 | 53 | 44 | 39 | 31 | 20 | 15 | 10 | 8 | 5 | 3 | 2 |
|  | Median | 100 | 80 | 62.5 | 55 | 49.5 | 41 | 30 | 23.5 | 17.5 | 13 | 9.5 | 6.5 | 4 |

Table 2

| Mixture | P  (MPa) | T  (°C) | *RD*_2520_  (mm) | *RD*_12600_  (mm) | *DS*  (times/mm) |
| --- | --- | --- | --- | --- | --- |
| AC-13 | 0.5 | 70 | 6.08 | 10.26 | 776 |
|  |  | 60 | 2.87 | 3.48 | 2369 |
|  |  | 50 | 2.17 | 2.58 | 5526 |
|  |  | 40 | 1.54 | 1.76 | 6843 |
|  |  | 30 | 1.11 | 1.28 | 11518 |
|  |  | 20 | 0.79 | 0.94 | 14533 |
|  | 0.7 | 70 | 8.09 | 12.64 | 672 |
|  |  | 60 | 4.06 | 4.93 | 1981 |
|  |  | 50 | 2.84 | 3.31 | 4627 |
|  |  | 40 | 1.75 | 2.10 | 6372 |
|  |  | 30 | 1.26 | 1.47 | 10090 |
|  |  | 20 | 0.86 | 1.14 | 13777 |
|  | 0.9 | 70 | 8.82 | 14.31 | 597 |
|  |  | 60 | 4.30 | 5.40 | 1721 |
|  |  | 50 | 3.12 | 3.80 | 3210 |
|  |  | 40 | 2.04 | 2.47 | 5546 |
|  |  | 30 | 1.38 | 1.63 | 9121 |
|  |  | 20 | 0.93 | 1.19 | 12857 |
|  | 1.1 | 70 | 9.75 | 15.82 | 551 |
|  |  | 60 | 5.07 | 7.14 | 1549 |
|  |  | 50 | 3.44 | 4.41 | 2745 |
|  |  | 40 | 2.21 | 2.75 | 4930 |
|  |  | 30 | 1.59 | 1.81 | 8186 |
|  |  | 20 | 0.98 | 1.29 | 11560 |
| AC-20 | 0.5 | 70 | 4.97 | 8.21 | 813 |
|  |  | 60 | 2.18 | 2.97 | 3651 |
|  |  | 50 | 1.62 | 2.11 | 5700 |
|  |  | 40 | 0.98 | 1.35 | 9460 |
|  |  | 30 | 0.56 | 0.73 | 13255 |
|  |  | 20 | 0.21 | 0.30 | 15867 |
|  | 0.7 | 70 | 7.04 | 11.11 | 773 |
|  |  | 60 | 2.90 | 4.13 | 2933 |
|  |  | 50 | 2.16 | 2.79 | 5132 |
|  |  | 40 | 1.22 | 1.62 | 8397 |
|  |  | 30 | 0.71 | 0.90 | 12054 |
|  |  | 20 | 0.35 | 0.52 | 14376 |
|  | 0.9 | 70 | 7.50 | 12.18 | 750 |
|  |  | 60 | 3.20 | 4.47 | 2243 |
|  |  | 50 | 2.14 | 3.17 | 4188 |
|  |  | 40 | 1.44 | 1.96 | 7067 |
|  |  | 30 | 0.76 | 1.05 | 11440 |
|  |  | 20 | 0.48 | 0.76 | 13743 |
|  | 1.1 | 70 | 8.46 | 13.61 | 642 |
|  |  | 60 | 3.53 | 5.69 | 2197 |
|  |  | 50 | 2.47 | 3.64 | 3684 |
|  |  | 40 | 1.65 | 2.32 | 6180 |
|  |  | 30 | 0.97 | 1.35 | 9772 |
|  |  | 20 | 0.59 | 0.81 | 12448 |
| ATB-30 | 0.5 | 70 | 3.56 | 6.64 | 1015 |
|  |  | 60 | 1.70 | 2.47 | 3352 |
|  |  | 50 | 1.38 | 1.88 | 5351 |
|  |  | 40 | 0.89 | 1.24 | 6470 |
|  |  | 30 | 0.46 | 0.61 | 8430 |
|  |  | 20 | 0.24 | 0.36 | 10702 |
|  | 0.7 | 70 | 4.52 | 7.59 | 1022 |
|  |  | 60 | 1.95 | 2.73 | 3171 |
|  |  | 50 | 1.48 | 1.99 | 3925 |
|  |  | 40 | 0.91 | 1.26 | 8832 |
|  |  | 30 | 0.48 | 0.63 | 11776 |
|  |  | 20 | 0.27 | 0.39 | 19022 |
|  | 0.9 | 70 | 5.12 | 8.19 | 985 |
|  |  | 60 | 2.08 | 2.78 | 3989 |
|  |  | 50 | 1.21 | 1.60 | 8832 |
|  |  | 40 | 0.69 | 0.89 | 9511 |
|  |  | 30 | 0.34 | 0.50 | 16488 |
|  |  | 20 | 0.18 | 0.28 | 82461 |
|  | 1.1 | 70 | 5.10 | 9.50 | 629 |
|  |  | 60 | 3.15 | 4.38 | 1781 |
|  |  | 50 | 2.02 | 3.07 | 3180 |
|  |  | 40 | 1.31 | 1.87 | 6547 |
|  |  | 30 | 0.67 | 0.95 | 7949 |
|  |  | 20 | 0.39 | 0.66 | 13092 |

Table 3

| Indicator | Mixture | Factor | SS | MS | F-value | P-value |
| --- | --- | --- | --- | --- | --- | --- |
| DS | AC-13 | T (°C) | 4.59×10^8^ | 9.18×10^7^ | 290 | 2.26×10^-14^ |
|  |  | P (MPa) | 1.38×10^7^ | 4.59×10^6^ | 15 | 1.05×10^-4^ |
|  | AC-20 | T (°C) | 5.40×10^8^ | 1.08×10^8^ | 317 | 1.18×10^-14^ |
|  |  | P (MPa) | 1.74×10^7^ | 5.81×10^6^ | 17 | 4.23×10^-5^ |
|  | ATB-30 | T (°C) | 6.35×10^9^ | 1.59×10^9^ | 53 | 1.63×10^-7^ |
|  |  | P (MPa) | 2.36×10^9^ | 7.87×10^8^ | 26 | 1.51×10^-5^ |
| RD | AC-13 | T (°C) | 413 | 83 | 106 | 3.60×10^-11^ |
|  |  | P (MPa) | 15 | 5 | 6 | 5.32×10^-3^ |
|  | AC-20 | T (°C) | 315 | 63 | 100 | 5.73×10^-11^ |
|  |  | P (MPa) | 12 | 4 | 6 | 5.23×10^-3^ |
|  | ATB-30 | T (°C) | 2.5 | 0.6 | 62.5 | 6.19×10^-8^ |
|  |  | P (MPa) | 0.8 | 0.3 | 25.6 | 1.68×10^-5^ |

**Table 4**

| Mixture | Test No. | P  (MPa) | T  (°C) | *RD*_2520_  (mm) | *CSI*  (times/mm^2^) |
| --- | --- | --- | --- | --- | --- |
| AC-13 | 1 | 0.7 | 60 | 3.907 | 1163 |
|  | 2 |  |  | 3.741 | 2816 |
|  | 3 |  |  | 3.496 | 3169 |
|  | 1 | 0.9 | 40 | 2.047 | 3186 |
|  | 2 |  |  | 1.988 | 3950 |
|  | 3 |  |  | 1.841 | 3118 |
| AC-20 | 1 | 0.7 | 60 | 2.786 | 1301 |
|  | 2 |  |  | 2.563 | 2576 |
|  | 3 |  |  | 2.696 | 2605 |
|  | 1 | 0.9 | 40 | 1.493 | 3640 |
|  | 2 |  |  | 1.370 | 5279 |
|  | 3 |  |  | 1.456 | 11550 |
| ATB-30 | 1 | 0.7 | 60 | 0.733 | 14163 |
|  | 2 |  |  | 0.835 | 17036 |
|  | 3 |  |  | 0.750 | 26615 |
|  | 1 | 0.9 | 40 | 0.537 | 33329 |
|  | 2 |  |  | 0.594 | 15829 |
|  | 3 |  |  | 0.566 | 53651 |
